# Immune Checkpoint Inhibitors: Sculpting the Landscape of Endometrial Cancer Treatment

**DOI:** 10.3390/cancers18213431

**Published:** 2026-10-23

**Authors:** Allan B. Huang, Lakeisha Mulugeta-Gordon, Dimitrios Nasioudis, Janos L. Tanyi

**Affiliations:** Division of Gynecologic Oncology, Department of Obstetrics and Gynecology, University of Pennsylvania Perelman School of Medicine, Philadelphia, PA 19104, USA; allan.huang@pennmedicine.upenn.edu (A.B.H.); lakeisha_mulugeta-gordon@med.unc.edu (L.M.-G.);

**Keywords:** endometrial cancer, immune checkpoint inhibitors, immunotherapy, mismatch repair deficiency, microsatellite instability, molecular classification, PD-1, PD-L1, CTLA-4, tumor microenvironment

## Abstract

**Background:** Outcomes for advanced or recurrent endometrial cancer (EC) remain poor, with historically limited systemic options beyond platinum-based chemotherapy. EC is uniquely immunotherapy-amenable among solid tumors: it harbors the highest prevalence of mismatch repair deficiency (dMMR) and microsatellite instability-high (MSI-H) status of any cancer, and its molecular classification into four major molecular subtypes defines a spectrum of tumor mutational burden, neoantigen load, and immune infiltration that predicts the response to checkpoint blockade. **Current Landscape:** Over the past several years, multiple programmed cell death protein 1 (PD-1) and programmed death-ligand 1 (PD-L1) inhibitors have received regulatory approval in EC, spanning monotherapy in dMMR/MSI-H disease, combination with carboplatin–paclitaxel in the first-line advanced setting, and combination with anti-angiogenic therapy in biomarker-unselected recurrent disease, fundamentally altering the standard of care. **Scope and Objectives:** This narrative review synthesizes the molecular rationale for checkpoint inhibition in EC, including the biological basis of its molecular subtypes and their immune microenvironments, and provides a focused overview of the trial evidence supporting each major checkpoint inhibitor class, including PD-1 inhibitors, PD-L1 inhibitors, cytotoxic T-lymphocyte-associated protein 4 inhibitors, and emerging targets. We further review the landscape of ongoing trials addressing unresolved questions, including chemotherapy-free regimens in dMMR disease, maintenance intensification for mismatch repair-proficient (pMMR) disease, neoadjuvant and fertility-sparing approaches, and novel combinations. This review aims to provide a comprehensive, up-to-date synthesis of this rapidly evolving field and to identify challenges and opportunities that will shape the next phase of immunotherapy in EC.

## Simple Summary

Endometrial cancer, which arises in the uterine lining, is the most common gynecologic cancer in developed countries, and outcomes for women whose disease has spread or returned have historically been poor. Immune checkpoint inhibitors are drugs that release the “brakes” on the immune system so it can recognize and attack cancer cells. Endometrial cancer is especially well suited to this approach because many tumors carry defects in DNA repair that produce large numbers of mutations, making them more visible to the immune system. In recent years, several of these drugs have been approved for endometrial cancer, alone or combined with chemotherapy or targeted therapy, changing the standard of care. This review explains how the molecular subtypes of endometrial cancer predict who benefits, summarizes the evidence for each drug class, and describes ongoing trials aimed at extending these benefits to more patients.

## 1. Introduction

Endometrial cancer (EC) is the most common gynecologic malignancy in developed countries and the fourth most frequent cancer affecting women overall, with an estimated 68,270 new cases and 14,450 deaths projected in 2026 in the United States alone [1,2]. Incidence and mortality continue to rise in parallel with aging populations and increasing rates of obesity, and EC represents the only gynecologic malignancy for which mortality has trended upward over recent decades [3]. The peak incidence occurs in the postmenopausal years, typically between 55 and 59 years of age, pointing to age as an independent risk factor for the disease [2].

EC incidence is strongly shaped by hormonal and metabolic risk factors. Obesity, through its promotion of unopposed estrogen excess, accounts for an estimated 50% of cases in developed countries and is associated with a markedly elevated risk of disease-related death [4]. Prolonged estrogen exposure through any mechanism, including early menarche, late menopause, nulliparity, or exogenous tamoxifen use, further amplifies risk in conjunction with metabolic risk factors [2,4]. Hereditary predisposition also plays an important role: Lynch syndrome, arising from germline mutations in mismatch repair (MMR) genes, confers a 40–60% lifetime risk of EC and accounts for approximately 4% of all cases [2,5].

Before the introduction of immune checkpoint inhibitors (ICIs), management of advanced and recurrent EC combined surgery, radiation, and systemic therapy, tailored to the disease extent, histology, and patient [4]. Surgery formed the foundation of treatment. In stage III disease, resection was followed by adjuvant chemotherapy and/or radiotherapy. In stage IV disease, systemic chemotherapy was the cornerstone, and surgery or radiotherapy was used mainly for symptom palliation and local control [6]. Notably, the benefit of cytoreduction and lymphadenectomy in advanced disease rests on retrospective series rather than randomized evidence [6]. Pelvic or whole-abdominal radiotherapy traditionally followed surgery for locally advanced disease. This approach controlled pelvic disease but did little to prevent distant relapse. In GOG-122, doxorubicin–cisplatin improved 5-year overall survival (OS) by 10 percentage points over whole-abdominal irradiation, shifting the standard toward chemotherapy [7]. GOG-258 later showed that adding radiation to chemotherapy in stage III–IVA disease reduced vaginal and nodal recurrence but did not prolong progression-free survival (PFS), as distant failure remained common [7].

Chemotherapy regimens were refined in parallel. GOG-177 showed that paclitaxel–doxorubicin–cisplatin (TAP) improved response and survival over doxorubicin–cisplatin, at the cost of considerable toxicity [8]. GOG-209 then established carboplatin–paclitaxel as the global first-line standard, demonstrating non-inferiority to TAP (median PFS 13 months; median OS 37 months) with better tolerability [8]. After platinum failure, no standard of care existed. In the control arm of KEYNOTE-775, doxorubicin or weekly paclitaxel yielded an objective response rate (ORR) of 14.7%, median PFS of 3.8 months, and median OS of 11.4 months [9]. Hormone-based regimens offered a less toxic alternative, but with modest activity: response rates of 22–24% and median PFS of 3.8–6.4 months in GOG-3007 [10]. Targeted therapy was largely limited to trastuzumab in human epidermal growth factor receptor 2 (HER2)-positive serous carcinoma, which extended PFS from 8.0 to 12.6 months when added to carboplatin–paclitaxel [11]. These modest outcomes defined the unmet need that ICIs would come to address.

EC has emerged as one of the most immunotherapy-amenable solid tumors, a property rooted in its intrinsic molecular biology. EC harbors the highest prevalence of MMR deficiency (dMMR) and microsatellite instability-high (MSI-H) status of any cancer, affecting approximately 25–30% of primary tumors [12,13]. These tumors generate a hypermutated phenotype with a high neoantigen burden, dense tumor-infiltrating lymphocyte (TIL) populations, and elevated programmed death-ligand 1 (PD-L1) expression that collectively render affected tumors highly susceptible to immune checkpoint blockade (ICB) [12,13]. More broadly, the molecular classification of EC, which is now anchored in the four subtypes defined by The Cancer Genome Atlas (TCGA) and formally incorporated into the 2023 International Federation of Gynecology and Obstetrics (FIGO) staging system, reveals a spectrum of immune microenvironments and mutational profiles that together define a biologically heterogeneous disease with variable, subtype-dependent responsiveness to immunotherapy [6,14,15].

The clinical translation of ICIs in EC has been rapid. Multiple programmed cell death protein 1 (PD-1) and PD-L1 inhibitors have received Food and Drug Administration (FDA) approval in EC over the past several years, spanning monotherapy in dMMR/MSI-H disease, combination with carboplatin–paclitaxel in the first-line advanced setting, and combination with anti-angiogenic therapy in biomarker-unselected second-line disease. These approvals have fundamentally altered how EC is managed, establishing MMR status [AH1.1] as an essential biomarker and ICI-based regimens as the new standard of care in advanced and recurrent disease. Yet, the growing number of approved agents, evolving biomarker-selection strategies, and expanding portfolio of ongoing trials have created an increasingly complex landscape for clinicians and researchers alike. A critical and comprehensive review of the evidence for immune checkpoint inhibition in EC, encompassing its molecular rationale, the clinical evidence across individual ICI classes, and the horizon of emerging strategies, is both timely and necessary. This review aims to synthesize that evidence and identify the challenges and opportunities that will define the next phase of EC immunotherapy.

## 2. Molecular Classification of Endometrial Carcinoma

The contemporary understanding of EC as a biologically heterogeneous disease was fundamentally reshaped by TCGA in 2013. Through comprehensive multiplatform genomic analysis of 373 endometrial carcinomas, TCGA identified four molecularly distinct subgroups: POLE ultramutated, microsatellite instability (MSI) hypermutated, copy-number low, and copy-number high. These molecular subgroups cut across traditional histomorphological boundaries and provided more biologically and prognostically meaningful tumor stratification than histology or grade alone [14]. A summary of the four major molecular subgroups of EC is provided in Table 1.

Clinical translation of the molecular subgroups was enabled by pragmatic classifiers, most notably ProMisE (Proactive Molecular Risk Classifier for Endometrial Cancer), which recapitulates the four TCGA subtypes using immunohistochemistry (IHC) for MMR proteins and p53, combined with targeted sequencing for POLE exonuclease domain mutations [16]. When applied to formalin-fixed, paraffin-embedded tissue, including pre-operative biopsies, ProMisE demonstrated near-perfect concordance between early diagnostic and final hysterectomy specimens (κ = 0.88), enabling molecular subtype assignment from the earliest point of diagnosis [16]. The TransPORTEC group independently confirmed the prognostic value of molecular subtyping in 423 participants of the PORTEC-3 trial, using next-generation sequencing to assess the POLE status and IHC to assess the p53 and MMR status [15,17]. The four subtypes, POLE-mutated (POLEmut), dMMR, no specific molecular profile (NSMP), and p53-abnormal (p53abn), have been incorporated into the World Health Organization (WHO) Classification of Tumors, 2023 FIGO staging system, and guidelines from the European Society of Gynaecological Oncology (ESGO), European Society for Radiotherapy and Oncology (ESTRO), and European Society of Pathology (ESP) [6,15]. Clinically, molecular testing follows a hierarchical stepwise algorithm: POLE sequencing is performed first, followed by MMR IHC, then p53 IHC, with NSMP assigned when all three are negative or normal [6].

A clinically important caveat is that approximately 3–6% of cases may harbor more than one molecular alteration (“multiple classifiers”) [18]. When a pathogenic POLE mutation co-exists with p53abn or dMMR status, current evidence supports classifying and treating such tumors according to the POLEmut subtype, which appears to confer the dominant prognostic effect [18]. On the other hand, in dMMR, p53abn multiple classifier tumors dMMR appears to be the dominant prognostic driver and should be treated as such [19].

### 2.1. POLE-Mutated (POLEmut)

The POLEmut subtype is defined by pathogenic somatic mutations in the exonuclease domain of DNA polymerase epsilon, generating an ultramutated phenotype exceeding 100 mutations per megabase and the highest tumor mutational burden (TMB) among all EC subtypes [14]. The two most common hotspot mutations, Pro286Arg and Val411Leu, were identified in 76% of ultramutated cases in the original TCGA cohort [14]. Pathogenic POLE exonuclease domain mutations are restricted to a defined list; non-pathogenic POLE variants should not be conflated with this favorable subtype [15]. POLEmut tumors account for 7–10% of all ECs, are predominantly endometrioid, are frequently high-grade with dense CD8+ TILs, and carry a five-year disease-specific survival exceeding 96% [14,15,16,20]. Their extreme immunogenicity, driven by a massive neoantigen burden and robust immunologic response, is believed to underlie this exceptional prognosis [20]. While recurrences do occur in POLEmut patients, data confirm that adjuvant therapy does not improve outcomes in early-stage POLEmut EC [AH2.1], providing biological rationale for de-escalation strategies studied in PORTEC-4a [AH3.1] and the RAINBO group of trials (expected completion in 2030, NCT05255653) [15,18,21].

### 2.2. MMR-Deficient (dMMR)

dMMR EC is defined by loss of one or more MMR proteins (MLH1, MSH2, MSH6, or PMS2) detectable by IHC, accounting for approximately 25–30% of all ECs [14,16,17]. The majority arise through somatic MLH1 promoter hypermethylation; approximately 15–17% are attributable to germline MMR mutations constituting Lynch syndrome, which accounts for roughly 4% of all EC [5,14,18]. Universal MMR/MSI screening of all newly diagnosed ECs is now recommended, serving the dual purpose of informing immunotherapy eligibility and identifying Lynch syndrome probands for cascade genetic testing [5]. Tumors with intact expression of all four MMR proteins, or a microsatellite-stable (MSS) status on molecular testing, are termed MMR-proficient (pMMR) and are discussed further in Section 2.5 [22].

dMMR tumors carry the second-highest TMB among EC subtypes and are characterized by high PD-L1 expression and elevated PD-1+ CD8+ TILs, rendering them highly susceptible to ICB [20]. Clinically, patients with dMMR tumors carry an intermediate prognosis, which is superior to p53abn but inferior to POLEmut [LM4.1] [14,17]. There is emerging data [AH5.1] suggesting that MLH1 loss is associated with worse outcomes and lower ICB response rates than Lynch syndrome-associated dMMR EC, potentially reflecting differences in biology and immune microenvironment composition amongst the dMMR group [15,23].

### 2.3. No Specific Molecular Profile (NSMP)

NSMP corresponds to the TCGA copy-number low group and is defined by the absence of pathogenic POLE mutations, intact MMR protein expression, and wild-type p53 IHC, comprising approximately 50% of population-based EC series [15,16]. These tumors are enriched for estrogen-receptor-positive low-grade endometrioid carcinomas with frequent phosphatase and tensin homolog (PTEN), PIK3CA, and CTNNB1 mutations, and carry a low TMB and somatic copy number alteration burden [14]. Notably, CTNNB1 (β-catenin) exon 3 mutations, detectable by next-generation sequencing, identify a subset with an elevated recurrence risk despite low-grade histology, leading to inclusion in the adjuvant treatment algorithm of PORTEC-4a as a marker of intermediate recurrence risk in the NSMP group [6,20,21]. Furthermore, Wnt/β-catenin pathway activation has been implicated in immune exclusion with potential interaction with immunotherapy combinations [6,20]. NSMP is the most heterogeneous subtype, encompassing aggressive histologies including clear-cell and dedifferentiated carcinoma alongside indolent low-grade tumors; sub-stratification using the histologic grade and estrogen receptor expression has been proposed to refine risk within this group [15].

### 2.4. p53-Abnormal (p53abn)

The p53abn subtype is defined by aberrant p53 protein expression on IHC, which includes overexpression, complete absence (null pattern), or cytoplasmic localization [18]. It accounts for 15–20% of all ECs but is responsible for more than 50% of EC mortality, with five-year disease-specific survival below 50% [15]. Accordingly, these tumors display a high propensity for intraperitoneal spread and distant metastases, as well as an elevated risk of recurrence after primary treatment. This subtype encompasses the majority (>90%) of uterine serous carcinomas and shares extensive genomic similarities with high-grade serous ovarian carcinoma, including recurrent amplifications of ERBB2, CCNE1, and FGFR3 [14]. Approximately 20–25% of cases overexpress HER2, and homologous recombination deficiency (HRD) is present in 20–46%, identifying targetable vulnerabilities relevant to subsequent sections [15,18]. All p53abn tumors with any myometrial invasion are classified as high risk under current ESGO/ESTRO/ESP guidelines and directed toward chemotherapy with or without radiation [15].

### 2.5. MMR-Proficient

Although not a molecular subtype in and of itself, MMR proficiency is the clinically operative category for immunotherapy selection, and dMMR versus pMMR status underpins the stratification of nearly all ICI trials discussed in Section 4. pMMR tumors retain intact MMR protein expression on IHC, or are MSS by polymerase chain reaction (PCR)- or sequencing-based testing, and comprise the remaining 70–75% of EC [13,22]. Biologically, the pMMR group consists predominantly of NSMP and p53abn tumors, which share a low TMB, sparse TILs, lower PD-L1 expression, and immunosuppressive gene expression signatures, rendering them immunologically “cold” [14,15,24]. POLEmut tumors are also MMR-proficient and MSS. However, their ultramutated, highly immunogenic phenotype is biologically distinct, so they should be considered separately from other pMMR tumors. Consistent with an immune-cold profile, ICI monotherapy yields ORRs of only 3–15% in pMMR EC [12,24]. Combination strategies therefore aim to enhance tumor immunogenicity. Chemotherapy induces immunogenic cell death and antigen presentation, while anti-angiogenic agents reverse vascular endothelial growth factor (VEGF)-mediated immunosuppression and improve T-cell infiltration. Combinations with poly(ADP-ribose) polymerase (PARP) inhibitors, which increase the TIL density, have also been examined [3,25].

### 2.6. Clinical Integration

Integration of molecular classification into guidelines has begun to meaningfully reshape EC management. PORTEC-4a addressed concerns that all patients with high/intermediate-risk EC receiving adjuvant brachytherapy were being overtreated, given effective salvage options. This randomized, open-label, phase 3, non-inferiority trial (n = 564) assigned patients 2:1 to molecular integrated risk profile-directed therapy (observation for favorable, brachytherapy for intermediate, pelvic radiotherapy for unfavorable profiles) versus standard brachytherapy. Molecular profile-based treatment was non-inferior for 5-year vaginal recurrence (4.5% vs. 1.6%), sparing 46% of patients adjuvant therapy without compromising survival, while suggesting improved locoregional control with pelvic radiotherapy in unfavorable profile patients. Toxicity was similar between groups.

Application of revised risk groups based on the molecular subtype to retrospective cohorts reveals that molecular subtyping [AH6.1] would have altered management in approximately 10–11% of all EC patients, identifying a third of POLEmut patients as potentially overtreated and nearly half of p53abn patients as potentially undertreated based on prior histology-based stratification [15]. The 2023 FIGO staging system further incorporates molecular features, producing significant stage migration, particularly for POLEmut (downstaging) and p53abn (upstaging) tumors [6]. The MMR status now drives FDA and European Medicines Agency (EMA) approvals of pembrolizumab and dostarlimab, and the predictive implications of the molecular subtype for the ICB response are explored in depth in subsequent sections.

## 3. Rationale for Immunotherapy in Endometrial Cancer

Endometrial cancer occupies a privileged position among solid tumors in terms of its susceptibility to immune-based treatment. Several intrinsic biological characteristics including a high prevalence of dMMR, elevated TMB, association with TILs, and expression of co-inhibitory checkpoints, including PD-1/PD-L1 and cytotoxic T-lymphocyte-associated protein 4 (CTLA-4), collectively underpin the rationale for deploying ICIs in this disease [24,26,27].

### 3.1. Immune Characteristics of Endometrial Cancer

The immune system’s ability to recognize and eliminate tumors depends on the generation of tumor-specific neoantigens, their presentation to cytotoxic T lymphocytes, and sustained activation of anti-tumor effector responses [26]. EC is particularly well-positioned to benefit from immunotherapy because many tumors harbor genomic features that amplify each of these steps [28,29]. In a genomic analysis of 30 different tumors, endometrial carcinomas ranked among the cancers with the highest somatic mutation burden, though the range of TMB was wide, from 1 to greater than 400 mutations per megabase. Other tumors with similarly high TMB include melanoma, non-small-cell lung cancer, and colorectal cancer, tumors that are paradigmatically responsive to checkpoint blockade [29]. This variation is strongly patterned by the molecular subtype: POLEmut tumors carry TMB exceeding 232 × 10^−6^ mutations/Mb and generate the greatest neoepitope burden of all EC subtypes, while dMMR/MSI-H tumors carry approximately 18 × 10^−6^ mutations/Mb. By contrast, copy-number low (NSMP) and copy-number high (p53abn) tumors are associated with a low TMB of 2.3 to 2.9 × 10^−6^ mutations/Mb and limited neoantigen production [14,24,26,29]. The immunological consequences of a high TMB combined with dense CD8+ TILs [LM7.1] and upregulation of immune checkpoint molecules are most pronounced in POLEmut and dMMR/MSI-H tumors, providing the mechanistic basis for ICI sensitivity in these subgroups [20,29].

Beyond mutational burden, EC cells engage in active immune modulation and crosstalk with their tumor microenvironment (TME). EC cells express indoleamine 2,3-dioxygenase 1 (IDO1), which converts L-tryptophan into the immunosuppressive metabolite L-kynurenine [30]. L-kynurenine drives regulatory T-cell (Treg) differentiation, promotes myeloid-derived suppressor cells (MDSCs), and is associated with decreased CD8+ and natural killer (NK) cell density within tumors [30]. EC cells also overexpress vascular endothelial growth factor receptor (VEGFR), fibroblast growth factor receptor (FGFR), and REarranged during Transfection (RET), sustaining an immunosuppressive, pro-angiogenic TME, supporting the mechanistic rationale for combining ICIs with anti-angiogenic agents such as lenvatinib [30,31]. Alongside these suppressive mechanisms, the high intrinsic immunogenicity of many EC tumors means that pre-existing anti-tumor immune responses are often present but held in check by inhibitory signals, a state amenable to reversal by checkpoint blockade.

### 3.2. Prevalence of MMR Deficiency and PD-L1 Expression

Among all human cancers, EC has the highest prevalence of dMMR/MSI-H status, a feature fundamental to its immunotherapy candidacy [12]. Approximately 25–30% of primary ECs are classified as dMMR/MSI-H, with estimates from recurrent disease ranging from 13% to 30% [12,24,27]. dMMR arises most commonly through somatic MLH1 promoter hypermethylation, while germline mutations in MMR genes (MLH1, MSH2, MSH6, PMS2) account for Lynch syndrome in approximately 4% of cases [5,31,32]. Regardless of the mechanism, MMR protein loss leads to the accumulation of replication errors at microsatellite sequences, generating a hypermutated phenotype with a high neoantigen load and heightened immune visibility [13,27]. EC has consistently demonstrated a high sensitivity to ICB across dMMR solid tumor types in basket trials, with an ORR of 48% and prolonged duration of response (DOR) after single-agent pembrolizumab in the KEYNOTE-158 cohort [33].

PD-1 (CD279) is a co-inhibitory surface receptor expressed on activated T cells, B cells, NK cells, macrophages, and dendritic cells [13,24]. Its primary ligand, PD-L1 (CD274), is constitutively expressed on antigen-presenting cells and is upregulated by inflammatory signals, most prominently interferon-gamma (IFN-γ), on both immune cells and cancer cells [13,27]. While the PD-1/PD-L1 axis normally functions as a negative feedback loop to prevent autoimmunity, in the context of PD-L1-expressing cancers, it is exploited to evade cytotoxic T-cell killing by delivering inhibitory signals via PD-1, attenuating effector function and promoting immune tolerance [25]. The PD-1/PD-L1 axis has consequently emerged as the most clinically relevant ICI target in EC. Overexpression of PD-1 and PD-L1 is common in EC across histotypes, with reported rates of 40–80% in endometrioid, 10–68% in serous, and 23–69% in clear-cell tumors [27].

### 3.3. The PD-1/PD-L1 Axis and Its Association with MMR Status

The relationship between the MMR status and PD-L1 expression strengthens the biological rationale for broader ICI use across EC, particularly in combination regimens. In a 2025 systematic review of ten studies encompassing 3486 patients, De Tommasi et al. found PD-L1 positivity significantly more prevalent in dMMR/MSI-H tumors (49%, 95% confidence interval [CI] 45–53%) than in pMMR/MSS tumors (33.5%, 95% CI 31.5–35.5%; *p* < 0.0001) [13]. Among pMMR/MSS tumors further stratified by molecular subtype, PD-L1 positivity was highest in POLEmut tumors (76.6%), followed by p53abn (39%) and NSMP tumors (31.9%) [13].

The preferential PD-L1 upregulation in dMMR tumors arises from its complex interplay with the adaptive immune response: interaction between highly immunogenic dMMR tumor cells and CD8+ T cells leads to the production of cytokines such as IFN-γ, IL-4, and IL-10, which have been implicated in upregulation of PD-L1 on tumor cells as an escape mechanism [22,27]. The biological basis for this association lies in the mutational consequences of MMR loss. When mismatch repair fails, replication errors accumulate at microsatellite sequences, and the resulting insertions and deletions shift the reading frame of coding genes [34]. These frameshift mutations generate abnormal peptides that the immune system recognizes as foreign, so dMMR tumors carry a substantially larger neoantigen burden than MSS tumors [34]. This antigenic load recruits cytotoxic T cells, and hypermutated ECs are densely infiltrated by CD3+ and CD8+ lymphocytes [34]. The tumor microenvironment responds to this sustained immune attack by upregulating inhibitory checkpoints: PD-1 is overexpressed on infiltrating lymphocytes, and PD-L1 is enriched on surrounding immune cells [34]. Notably, PD-L1 on tumor cells themselves does not differ appreciably by MMR status, suggesting that PD-L1 enrichment in dMMR EC resides largely within the immune compartment [34]. PD-L1 expression in dMMR EC is therefore best understood as an adaptive response to an inflamed tumor, not a constitutive tumor-intrinsic trait. Checkpoint blockade releases this counterbalancing brake on an anti-tumor immune response that is already present [34].

PD-L1 expression is further regulated at the epigenetic, transcriptional, and translational levels, contributing to expression heterogeneity across and within molecular subtypes [25]. Discordance between PD-L1 expression in primary tumors and matched metastatic lesions has also been documented, complicating its use as a standalone patient selection biomarker. In the context of EC, MMR status remains the most relevant predictive biomarker of ICI efficacy due to its role as a driver of derangements in the PD-1/PD-L1 axis.

### 3.4. The CTLA-4 Axis

CTLA-4 is a checkpoint that acts earlier in the anti-tumor immune response than PD-1/PD-L1. Naïve T-cell activation requires T-cell receptor engagement plus a co-stimulatory signal, delivered when CD28 binds the B7 ligands CD80/CD86 on antigen-presenting cells. CTLA-4, expressed on activated T cells and Tregs, competes with CD28 for these ligands and attenuates co-stimulation [26]. PD-1, by contrast, is engaged by PD-L1 and programmed death-ligand 2 (PD-L2) within the TME and dampens the activity of already-primed effector T cells [26]. CTLA-4 blockade therefore primarily enhances T-cell priming and targets Tregs. PD-1/PD-L1 blockade releases restraint on tumor-infiltrating effector cells. These complementary mechanisms underpin combined CTLA-4/PD-1 blockade, already approved in dMMR colorectal [31].

Data linking CTLA-4 to the MMR status and molecular subtype in EC are limited. In TCGA, CTLA4 expression correlated strongly with CD8A across all subtypes. This is consistent with adaptive immune resistance in T-cell-inflamed tumors [35,36]. CTLA4 was significantly upregulated in POLEmut tumors alongside other exhaustion markers, but was essentially unchanged in MSI compared with MSS tumors [35]. Some reviews report higher CTLA-4 expression in MSI-H EC, but these data are partly extrapolated from colorectal cancer [31]. In a cohort of 68 endometrioid and serous carcinomas, CTLA-4 expression was higher in p53-mutant tumors (high IHC expression in 72% vs. 28% of p53 wild-type) and associated with serous histology, higher grade, and advanced stage [37]. This study was small, however, and did not use integrated molecular classification. CTLA-4 has not been specifically characterized in NSMP EC, and its role in this largely immune-cold subtype remains unknown [24,29]. These gaps are mirrored by limited, largely MMR-unselected trial data for CTLA-4 inhibitors (Section 4.3), highlighting the need for biomarker-driven evaluation.

### 3.5. Tumor-Infiltrating Lymphocytes, Immunosuppressive Myeloid Cells, and the Tumor Immune Microenvironment

The density and composition of TILs form an informative biomarker of immune engagement and ICI responsiveness in EC. CD8+ TIL density in the peritumoral stroma has been shown to be an independent prognostic factor for improved survival, underscoring the value of immune infiltration metrics as a complementary biomarker [27]. TIL abundance is strongly patterned by the molecular subtype: dMMR/MSI-H tumors harbor significantly higher CD8+ infiltration and greater numbers of activated T cells and PD-L1-positive cells compared to MSS tumors, reflecting an active but checkpoint-suppressed anti-tumor immune state [27]. POLEmut tumors exhibit similarly prominent TIL infiltration with overexpression of cytotoxic markers (IFN-γ, perforin, granzyme B), consistent with their near-universal favorable prognosis and recurrence rates below 3% [20,27]. In contrast, p53abn and NSMP tumors display low immune infiltration and high immunosuppressive gene expression, rendering them immune ‘cold’ and less responsive to ICI monotherapy [24,29]. MDSCs and tumor-associated macrophages (TAMs) also uniquely shape the TME towards a pro-tumorigenic state. In EC, PTEN is the most commonly mutated tumor suppressor gene and has been associated with a preponderance of Tregs, MDSCs and immunosuppressive M2 macrophages in the TME [22]. The diversity of players in the TME of EC offers a rich variety of potential immunotherapy targets (many of which will be discussed in subsequent sections of this review), strengthening the rationale for ICI in this tumor type.

Altogether, the immune landscape of EC is characterized by a high TMB and neoantigen burden, particularly in the dMMR and POLEmut subtypes, as well as elevated PD-L1 expression, abundant TILs, and an active but checkpoint-restrained anti-tumor immune response. These mechanisms provide a compelling biological foundation for ICI-based therapy. The ‘hot’ versus ‘cold’ tumor dichotomy, shaped by the interplay of TILs, TAMs, MDSCs, and checkpoint molecule expression, offers a practical framework for predicting the ICI response and identifying patients who may require combination strategies to achieve meaningful clinical benefit.

## 4. Main Immune Checkpoint Inhibitor Groups

The following section is a narrative overview of the major classes of ICI therapies and a focused synthesis of trial evidence supporting therapies within each class. Table 2 provides a summary of FDA-approved ICI therapies in EC, the major efficacy data supporting approval, and regulatory approval dates. This scope is expanded in Table 3, which summarizes key details of major trials examining ICI in EC, regardless of FDA approval status.

### 4.1. PD-1 Inhibitors: Pembrolizumab, Nivolumab, Dostarlimab

#### 4.1.1. Pembrolizumab

Pembrolizumab (Keytruda, Merck) is a humanized IgG4 monoclonal antibody that competitively blocks the PD-1 receptor on T lymphocytes. By disrupting PD-1 interaction with both of its ligands, PD-L1 and PD-L2, it prevents downstream immune suppression and restores cytotoxic T-cell surveillance and anti-tumor immune activity [39]. In EC, the rationale for PD-1 blockade is particularly compelling in dMMR and MSI-H tumors, which carry a high neoantigen burden, elevated tumor mutational load, and increased PD-1/PD-L1 expression relative to pMMR tumors [33].

##### Monotherapy

The phase II multicohort basket trial KEYNOTE-158 provided the pivotal evidence for pembrolizumab monotherapy in this setting. Among 79 evaluable EC patients with previously treated advanced dMMR/MSI-H EC, pembrolizumab 200 mg every three weeks yielded an ORR of 48% (95% CI, 37–60), including a 14% complete response rate, after a median follow-up of 42.6 months. Median DOR was not reached, with an estimated 68% of responses sustained at three years. Median PFS was 13.1 months and median OS was not reached, with 3-year OS and PFS estimates at 60% and 37% respectively [33]. The most common treatment-related toxicities included pruritus (24%), fatigue (21%), and diarrhea (16%) [33]. Based on these results, the FDA granted pembrolizumab monotherapy accelerated approval in 2017 for unresectable or metastatic dMMR/MSI-H solid tumors following prior therapy [33]. A separate, EC-specific approval followed in March 2022 based on KEYNOTE-158 [40]. A key limitation of KEYNOTE-158 was its single-arm design, precluding direct comparative efficacy assessment; additionally, the majority of patients had their PD-L1 status not assessed, limiting conclusions about PD-L1 as a predictive biomarker in this context.

##### Combination with Chemotherapy

The phase III KEYNOTE-868/NRG-GY018 trial evaluated the addition of pembrolizumab to carboplatin–paclitaxel followed by pembrolizumab maintenance therapy in patients with advanced or recurrent EC of any histologic subtype, excluding carcinosarcoma, stratified by MMR status. Importantly, all patients were required to have measurable disease by Response Evaluation Criteria in Solid Tumors (RECIST) 1.1, except for stage IVB patients. Prior radiotherapy and/or hormonal therapy was permitted. In the dMMR cohort, the 12-month PFS rate was 74% in the pembrolizumab arm versus 38% in the placebo arm (hazard ratio [HR] 0.30, 95% CI 0.19–0.48; *p* < 0.001 [AH9.1]), representing a 70% reduction in the risk of progression or death. A clinically meaningful, though smaller, benefit was also seen in the pMMR cohort, with a median PFS of 13.1 versus 8.7 months (HR 0.54, 95% CI 0.41–0.71; *p* < 0.001) [39,41]. These results supported FDA approval of pembrolizumab in combination with carboplatin–paclitaxel followed by pembrolizumab maintenance for primary advanced or recurrent EC, regardless of MMR status, in June 2023. In an updated OS analysis of NRG-GY018, at the 48-month assessment of dMMR patients, 80% were alive in the pembrolizumab arm vs. 60% in the placebo arm (HR 0.53, 95% CI 0.32–0.89). In the pMMR group, an OS benefit of 11.8 months was seen in the pembrolizumab arm, but this did not meet statistical significance (46.9 vs. 35.1 months, HR 0.84, 95% CI 0.66 to 1.06). However, it is important to note that a numerical benefit was seen despite 57% of the pMMR patients receiving post-study ICI [42].

The ENGOT-en11/GOG-3053/KEYNOTE-B21 phase III trial examined whether adding pembrolizumab to adjuvant carboplatin–paclitaxel (with or without radiotherapy) improved disease-free survival (DFS) in 1095 patients with newly diagnosed high-risk EC who were disease-free after curative-intent surgery. In the intention-to-treat population, pembrolizumab did not improve DFS (HR 1.02, 95% CI 0.79–1.32; *p* = 0.570), with similar 2-year estimated DFS rates in pembrolizumab vs. placebo groups (75% versus 76%) [43]. However, a pre-planned subgroup analysis of the dMMR population (n = 281) demonstrated a clinically meaningful DFS benefit favoring pembrolizumab (HR 0.31, 95% CI 0.14–0.69), whereas no benefit was observed in the pMMR subgroup (HR 1.20, 95% CI 0.91–1.57) [43]. The absence of radiologically apparent residual disease at enrollment is a key distinction from the first-line advanced/recurrent disease trial NRG-GY018 and is hypothesized to have contributed to the null result in the overall population, as a sufficient tumor antigen burden may be required to stimulate a more robust anti-tumor immune response, particularly in less immunogenic pMMR disease [43]. These results confirmed the importance of appropriate EC patient selection for ICI therapy on the basis of biologically rational biomarkers. POLEmut patients were excluded from the trial, limiting conclusions in that subgroup.

##### Combination with Targeted Therapy

Lenvatinib (Lenvima, Eisai) is an oral multi-kinase inhibitor targeting a wide range of angiogenic growth factors, most notably VEGFR1–3; it has also demonstrated activity against FGFR1–4, RET, KIT, and platelet-derived growth factor receptor alpha (PDGFRα). In pre-clinical models, it depletes immunosuppressive TAMs and expands intratumoral CD8+ T cells, potentiating PD-1 blockade [22].

Following promising efficacy results from the phase IB/II KEYNOTE-146, the phase III KEYNOTE-775 trial randomized 827 patients with progressive disease following one prior platinum-based regimen 1:1 to lenvatinib plus pembrolizumab or physician’s choice chemotherapy (doxorubicin or weekly paclitaxel), regardless of MMR status. No prior exposure to anti-PD-1 or anti-VEGF therapy was allowed. The combination significantly improved PFS, OS, and ORR. At 5-year follow-up (median 68.8 months), 5-year OS rates were 16.7% versus 7.3% in the pMMR population, 36.5% versus 9.8% in the dMMR subgroup, and 19.9% versus 7.7% in the overall cohort. Median OS was 18.0 versus 12.2 months in the pMMR population (HR 0.70, 95% CI 0.60–0.83) and 31.9 versus 8.6 months in the dMMR subgroup (HR 0.44, 95% CI 0.29–0.67). ORR was 32.4% versus 14.8% and 41.5% versus 12.3% in pMMR and dMMR populations respectively [44]. Lenvatinib dose reductions were required in 68.7% of participants, most commonly for hypertension and diarrhea, though no new safety signals emerged over 5 years of follow-up [44]. Although the regimen demonstrated efficacy in all-comers, the FDA limited approval to patients with pMMR tumors given the availability of single-agent ICI options (pembrolizumab or dostarlimab) that are highly effective in dMMR disease without the additional toxicity profile of lenvatinib. The EMA approval remains in place without restriction on MMR status. Based on these data, the combination was established as a standard second-line option for pMMR patients.

LEAP-001 tested whether lenvatinib plus pembrolizumab could replace chemotherapy in the first-line setting, randomizing 842 patients with stage III–IV or recurrent EC 1:1 to the combination or carboplatin–paclitaxel. Eligible patients had received no prior chemotherapy or one prior line of platinum-based chemotherapy in the neoadjuvant or adjuvant setting, with a minimum disease-free interval of ≥6 months. The trial did not meet its primary endpoints: median PFS was 9.6 versus 10.2 months in the pMMR population (HR 0.99[AH10.1]) and median OS was 30.9 versus 29.4 months (HR 1.02; non-inferiority *p* = 0.246) [45]. In the dMMR population, PFS and OS were improved in the lenvatinib plus pembrolizumab arm compared to chemotherapy (PFS HR 0.61, OS HR 0.57) [45]. While these exploratory dMMR subgroup findings reached statistical significance, the study was powered to detect differences in the pMMR group. A signal favoring the combination was observed in the approximately 14% of patients with prior neoadjuvant or adjuvant chemotherapy, consistent with its established post-platinum activity from KEYNOTE-775 [45]. These results confirm that lenvatinib plus pembrolizumab does not supplant carboplatin–paclitaxel alone as frontline standard of care, but outcomes in the dMMR subgroup raise questions about whether lenvatinib plus pembrolizumab may prove to be more efficacious in a biomarker-selected population.

Mirvetuximab soravtansine (Elahere, AbbVie), a folate receptor alpha (FOLR1)-targeting antibody–drug conjugate (ADC), was combined with pembrolizumab in an investigator-initiated, single-arm phase II trial (NCT03835819) of recurrent or persistent pMMR/MSS serous EC requiring FOLR1 2+ expression in ≥50% of cells and one to four prior therapy lines. Of the planned sample size of 35 patients, 18 were enrolled before the trial closed early for slow accrual; closure reflected feasibility, not futility, as the regimen had already met its Stage 1 continuation criteria of two objective responses. The confirmed ORR was 28% (95% CI, 10–53%; one complete and four partial responses), PFS at 6 months was 24.4% and median DOR of 8.17 months [46]. All five confirmed responses occurred in patients without prior ICI exposure; the most common treatment-related toxicities were diarrhea (44%), fatigue (44%), and liver enzyme elevation (44%), with no grade 4–5 toxicities noted [46]. A composite biomarker score combining FOLR1 expression and immune-cell density correlated with the response, though this remains hypothesis-generating [46]. While the combination of mirvetuximab soravtansine and pembrolizumab showed promising clinical activity in patients with unfavorable serous histology, the small cohort and early closure limit its translation into clinical standard of care.

#### 4.1.2. Nivolumab

Nivolumab (Opdivo, Bristol Myers Squibb) is a monoclonal antibody that selectively binds the PD-1 receptor, similarly blocking PD-L1 and PD-L2 engagement and restoring T-cell anti-tumor activity. In EC, nivolumab has not received FDA approval as a standalone indication but has demonstrated activity primarily in dMMR/MSI-H and hypermutated tumors.

##### Monotherapy

Evidence from the basket trial NCI-MATCH (EAY131) Arm Z1D-A showed nivolumab to be effective in dMMR non-colorectal cancers, including endometrial carcinomas and carcinosarcoma (combined n = 17), with activity consistent with that seen for other PD-1 inhibitors in this molecularly selected population [47]. A phase II Japanese multicohort study by Tamura et al. examined nivolumab monotherapy in gynecologic cancers; the biomarker unselected uterine cancer cohort demonstrated an ORR of approximately 23% and a 6-month OS rate of 73% [48,49]. This selection-dependent pattern was corroborated by a phase II trial from Friedman et al., which restricted enrollment to patients with dMMR/MSI-H recurrent endometrial or ovarian cancer (83% endometrioid endometrial histology); nivolumab monotherapy achieved an ORR of 58.8% in the evaluable cohort, including 7 complete and 13 partial responses. Notably, 64.7% of patients remained progression-free at 24 weeks [50]. The trial met its two primary endpoints (ORR and PFS at 24 weeks) early. Taken together, these studies suggest that nivolumab’s single-agent activity in EC depends heavily on dMMR/MSI-H selection, with only modest efficacy in biomarker-unselected populations.

##### Combination with Targeted Therapy

Cabozantinib, a multitargeted tyrosine kinase inhibitor (TKI) targeting VEGFR2 and RET among other kinases, has been evaluated in combination with nivolumab as a strategy to overcome this limited single-agent activity. In the phase II trial by Lheureux et al., ICI-naïve patients with recurrent EC were randomized 2:1 to cabozantinib plus nivolumab (Arm A, n = 36) versus nivolumab monotherapy (Arm B, n = 18), stratified by microsatellite or MMR status. The combination arm achieved a median PFS of 5.3 months versus 1.9 months with nivolumab alone (HR 0.59; *p* = 0.09, met prespecified significance criteria of *p* = 0.10), with ORRs of 25% versus 11% and clinical benefit rates of 69% versus 22% [51]. Serious treatment-related adverse events occurred in 31% of the combination arm, including one fatal case of colonic perforation with sepsis. In an exploratory ICI rechallenge cohort (Arm C, n = 20) who had previously received anti-PD-(L)1 therapy, cabozantinib–nivolumab yielded a 25% ORR, similar to the ICI-naïve Arm A, warranting further investigation [51]. Of note, 10 of the 20 patients in Arm C had uterine carcinosarcoma, a rare histology typically less represented in trials. The combination remains investigational and unapproved in EC, though these findings reflect the underlying synergy between antiangiogenic and checkpoint-inhibitor strategies. The phase II NRG-GY025 trial is ongoing, comparing nivolumab monotherapy to nivolumab plus ipilimumab in dMMR recurrent EC [2].

#### 4.1.3. Dostarlimab

Dostarlimab (Jemperli, GlaxoSmithKline) is a humanized IgG4 monoclonal antibody that binds PD-1 with high affinity, blocking both PD-L1 and PD-L2 ligand engagement. Mechanistically, it is similar to pembrolizumab and nivolumab in binding primarily at the flexible loop regions of PD-1 rather than directly at the ligand-binding interface [40].

##### Monotherapy

The phase I GARNET trial established dostarlimab monotherapy as an effective second-line treatment in dMMR/MSI-H advanced or recurrent EC. In the final interim analysis of 143 evaluable patients with dMMR/MSI-H disease who had progressed on or after platinum-based chemotherapy (≤2 prior lines), dostarlimab administered at 500 mg every 3 weeks for four cycles, then 1000 mg every 6 weeks, achieved an ORR of 45.5% (95% CI, 37.1–54.0), a complete response rate of 16.1%, and a median DOR that was not reached after a median follow-up of 27.6 months [52]. The estimated probability of maintaining a response at 24 months was 83.7%, indicating highly durable responses in biomarker selected patients. In patients with pMMR/MSS disease, responses were less common (ORR 15.4%) but durable among responders (median DOR 19.4 months), highlighting the need for better biomarker selection in this group [52]. These data supported FDA accelerated approval of dostarlimab monotherapy in April 2021 for dMMR recurrent or advanced EC after platinum-containing therapy, and subsequent approval for dMMR advanced solid tumors more broadly in August 2021 [33,40]. Dostarlimab monotherapy was well tolerated, and only 8.6% of patients discontinued treatment due to toxicity. The most common adverse effects were fatigue (17.8%), diarrhea (14.6%), and nausea (13.7%), while immunotherapy-related toxicities included hypothyroidism (8.3%) and arthralgia (3.2%). An important limitation of GARNET was its single-arm design without a randomized comparator, and biomarker analyses beyond MMR status were post hoc and not powered for formal hypothesis testing [52].

##### Combination with Chemotherapy

The pivotal phase III RUBY trial (ENGOT-en6-NSGO/GOG-3031) randomized 494 patients with primary advanced (stage III–IV) or recurrent EC of any histology, including carcinosarcoma, 1:1 to carboplatin–paclitaxel plus dostarlimab or placebo, followed by dostarlimab or placebo maintenance for up to 3 years. Measurable disease was required for patients with stage IIIA to IIIC1 cancer, but not required for stage IIIC1 non-endometrioid histology or stage IIIC2 and beyond regardless of histology. In the dMMR/MSI-H population (approximately 24% of the overall cohort), dostarlimab reduced the risk of progression or death by 72% (HR 0.28, 95% CI 0.16–0.50; *p* < 0.001); estimated 24-month PFS was 61.4% versus 15.7% and 24-month OS was 83.3% versus 58.7%. In the overall population, 24-month PFS was 36.1% versus 18.1% (HR 0.64; *p* < 0.001) and 24-month OS was 71.3% versus 56.0% (HR 0.64, 95% CI 0.46–0.87) [53]. A PFS benefit was also observed in the pMMR/MSS subgroup (24-month PFS 28.4% vs. 18.8%; HR 0.76, 95% CI 0.59–0.98), though the OS difference in this subgroup did not reach statistical significance at initial analysis [53]. On the basis of OS benefit seen in the all-comer population RUBY, dostarlimab plus carboplatin–paclitaxel followed by dostarlimab maintenance garnered FDA approval for advanced or recurrent EC regardless of MMR status [40]. It should be noted that the RUBY trial enrolled a higher proportion of patients with recurrent disease (47.8%) than advanced primary disease, and subgroup analyses suggested heterogeneity of the PFS benefit—particularly in patients with stage III disease and those without measurable disease at baseline—which may partly reflect the limited follow-up time and sample size in these subgroups rather than a true lack of effect [53].

### 4.2. PD-L1 Inhibitors: Atezolizumab, Avelumab, Durvalumab

Unlike PD-1 inhibitors, which bind the inhibitory receptor on T lymphocytes, PD-L1 inhibitors target the ligand expressed on tumor cells and antigen-presenting cells, blocking its interaction with both PD-1 and CD80. This mechanistic distinction may theoretically preserve some physiological immune regulation and has been associated with a potentially reduced burden of immune-related adverse events [2]. In EC, PD-L1 inhibitors have been studied across the monotherapy and chemotherapy-combination settings, with efficacy patterns broadly mirroring those of PD-1 inhibitors (substantially greater in dMMR disease) though the approval landscape differs meaningfully between agents.

#### 4.2.1. Atezolizumab

Atezolizumab (Tecentriq, Genentech/Roche) is a humanized IgG1 monoclonal antibody targeting PD-L1. Early-phase single-agent data in advanced EC were modest: in the phase I expansion cohort of NCT01375842 (15 patients), the ORR was 13.3%, with responses largely confined to patients with MSI-H or heavily immune-infiltrated tumors, and a median PFS of 1.4–1.7 months [26,49].

##### Combination with Chemotherapy

The phase III AtTEnd trial (NCT03603184) was the first randomized study to evaluate atezolizumab in combination with carboplatin–paclitaxel in advanced or recurrent EC, enrolling 551 patients with endometrial carcinoma or carcinosarcoma who had received no prior systemic chemotherapy for recurrence (2:1 atezolizumab to placebo). In the dMMR population (approximately 23% of each arm), median PFS was not reached in the atezolizumab arm versus 6.9 months in the placebo arm (HR 0.36, 95% CI 0.23–0.57; *p* = 0.0005). In the overall population, a modest PFS benefit was seen, with median a PFS of 10.1 versus 8.9 months (HR 0.74, 95% CI 0.61–0.91; *p* = 0.022). Interim OS results showed a numerical advantage for atezolizumab (38.7 vs. 30.2 months; HR 0.82, 95% CI 0.63–1.07; *p* = 0.048), though the OS stopping boundary had not been crossed and the trial continues [54]. Crucially, unlike the RUBY and NRG-GY018 trials, no statistically significant PFS benefit was demonstrated in the pMMR subgroup in AtTEnd, a divergence that the authors note may partly reflect the higher proportion of Asian patients enrolled (approximately 20%), a population in whom responses to checkpoint inhibition may differ [54]. Atezolizumab has not received FDA or EMA approval for EC as of this writing, attributable to the immature OS data, lack of benefit in pMMR group, and an occupied clinical niche that already contains pembrolizumab and dostarlimab in the same treatment setting.

##### Combination with Targeted Therapy

The combination of atezolizumab with anti-VEGF therapy was studied in the phase II study by Fuh et al. (NCT03526432) evaluating atezolizumab plus bevacizumab in 56 evaluable patients with ICI-naïve recurrent EC after platinum-based chemotherapy, of whom 45 had pMMR disease. The ORR was 33.9%, with a median DOR of 16 months, median PFS of 8.8 months, and a notably prolonged median OS of 41.55 months. Grade 3–4 toxicities were led by hypertension (21.5%), fatigue (7.7%), and diarrhea (7.7%) [38]. These results are promising, particularly for the pMMR population; however, this study was reported as a meeting abstract at ASCO 2026 without a full peer-reviewed publication, and findings should be considered preliminary pending complete data disclosure.

#### 4.2.2. Avelumab

Avelumab (Bavencio, Pfizer/Merck KGaA), an anti-PD-L1 antibody, has been evaluated in EC through three sequential cohorts of a single investigator-initiated phase II platform trial (NCT02912572, Konstantinopoulos et al.): monotherapy, avelumab plus the PARP inhibitor, Talazoparib, and Avelumab plus the VEGFR1–3 inhibitor, Axitinib.

##### Monotherapy

In the monotherapy cohort, 31 patients, composed of 15 with dMMR and 16 with pMMR, with recurrent or persistent EC and at least one prior chemotherapy line were treated with avelumab; while POLEmut was allowed, no POLEmut patients were ultimately enrolled. The dMMR ORR was 26.7% (95% CI, 7.8–55.1%), with a PFS at 6 months of 40.0%; the pMMR cohort closed early for futility (ORR 6.25%). Toxicity was manageable, with no grade 4–5 treatment-related events [55]. An exploratory biomarker analyses showed that among those with known PD-L1 status, all objective responses occurred in PD-L1-negative patients, further emphasizing the primacy of the MMR status in predicting the ICI response.

##### Combination with Targeted Therapy

Given limited monotherapy activity in pMMR disease, avelumab was combined with talazoparib in 35 patients on the basis of pre-clinical evidence of synergy with PARP inhibitors. Patients in this cohort of the avelumab platform trial had no restrictions on prior lines of therapy or histology but were required to have measurable disease. The confirmed ORR was 11.4% (95% CI, 3.2–26.7%), and PFS at 6 months was 22.9%, with manageable, class-consistent hematologic toxicity (anemia, thrombocytopenia, neutropenia) [56]. The approximately 20% of patients with HRD tumors derived a significantly greater clinical benefit (83.3% vs. 17.4%; *p* = 0.01). The subset of patients with both HRD tumors and a platinum-free interval of ≥6 months achieved an ORR of 25% and PFS at 6 months of 58.3%. PD-L1 status was not correlated with a clinical benefit [56].

Most recently, a third cohort combined avelumab with axitinib, a more potent and VEGFR-selective TKI than lenvatinib, in 35 ICI-naïve pMMR patients without restriction on prior lines of therapy. The ORR was 40%, with a median PFS of 7 months and PFS at 6 months of 55.8% [57]. Efficacy was higher in p53-mutated tumors (78.1% of the cohort; ORR 48%) than NSMP tumors (ORR 28.6%), attributed to TP53-driven VEGFR hyperactivation and an immunosuppressive, Treg-rich microenvironment reversible by antiangiogenic therapy. Hypertension, the expected on-target effect of VEGFR inhibition, was the principal grade 3 toxicity, occurring in 37% of patients, with no unexpected safety signals [57].

Avelumab has not advanced to phase III or gained regulatory approval in EC. Unlike durvalumab and atezolizumab, its evidence base remains entirely in uncontrolled phase II data; both combination strategies nonetheless met prespecified efficacy thresholds, and the axitinib combination showed activity comparable to the approved lenvatinib/pembrolizumab regimen in a more heavily pretreated population.

#### 4.2.3. Durvalumab

Durvalumab (Imfinzi, AstraZeneca) is a selective monoclonal antibody that binds PD-L1. Single-agent activity was established in the phase II PHAEDRA trial (ANZGOG1601; NCT03015129), which enrolled 71 patients with advanced EC in dMMR (n = 36) and pMMR (n = 35) cohorts receiving durvalumab 1500 mg intravenously every 4 weeks. In the dMMR cohort, the ORR by RECIST was 47% (95% CI, 32–63%), with a median PFS of 8.3 months and a 12-month OS rate of 71%; responses were notably higher in chemotherapy-naïve patients (57%) than in those with one prior line (38%). In the pMMR cohort, durvalumab had limited efficacy; the ORR was 3%, with a median PFS of 1.8 months. Immune-related adverse events were predominantly grade 1–2, with only three patients discontinuing treatment due to a treatment-related event [58].

The phase III DUO-E trial (NCT04269200) randomly assigned 718 patients with newly diagnosed advanced or recurrent EC (uterine sarcomas excluded) 1:1:1 to carboplatin–paclitaxel alone, carboplatin–paclitaxel plus durvalumab followed by maintenance durvalumab, or the same regimen with maintenance durvalumab plus olaparib. Both experimental arms met their primary PFS endpoints versus chemotherapy alone (durvalumab: HR 0.71, 95% CI 0.57–0.89; *p* = 0.003; durvalumab plus olaparib: HR 0.55, 95% CI 0.43–0.69; *p* < 0.0001), with a PFS benefit observed in both dMMR and pMMR exploratory subgroups and interim OS results supportive of a benefit in the experimental arms. The incremental benefit of olaparib beyond durvalumab alone appeared concentrated in the pMMR and PD-L1-positive populations, with little additive effect in dMMR disease [59]. Anemia was more frequent in the durvalumab + olaparib arm, but there were overall similar rates of treatment discontinuation and grade 3 or higher adverse events across arms [59]. Based on DUO-E, durvalumab plus carboplatin–paclitaxel received FDA approval for dMMR primary advanced or recurrent EC in 2024; the durvalumab plus olaparib maintenance combination has yet to garner approval as the PFS benefit seen in pMMR patients was part of an exploratory analysis and OS data continues to mature [2].

### 4.3. CTLA-4 Inhibitors: Tremelimumab and Ipilimumab

As outlined in Section 3.4, CTLA-4 blockade acts primarily at the level of T-cell priming and Treg-mediated suppression, a mechanism distinct from and potentially complementary to PD-1/PD-L1 blockade [26]. This rationale has driven investigation of CTLA-4 inhibitors in EC, both as monotherapy and in combination with PD-1/PD-L1 agents. However, EC-specific data remain sparse, limited to early-phase studies, and no CTLA-4 inhibitor currently holds regulatory approval in this indication.

#### 4.3.1. Tremelimumab

Tremelimumab is a human IgG2 kappa monoclonal antibody targeting CTLA-4. Its most clinically informative evaluation in EC comes from the randomized phase II Rubinstein et al. study (NCT03015129), which compared durvalumab monotherapy to durvalumab plus tremelimumab every 4 weeks for four cycles followed by durvalumab alone in 75 evaluable patients with recurrent endometrial carcinoma or carcinosarcoma following at least one prior platinum-based regimen. The population was predominantly pMMR (approximately 82% in each arm). Neither arm met prespecified efficacy thresholds: the ORR was 10.8% for durvalumab alone and 5.3% for the combination, with both confirmed complete responses occurring in dMMR patients. Median PFS was 7.4 and 7.9 weeks respectively. The combination arm generated more frequent immune-mediated adverse events, notably colitis (10% grade ≥3, no colitis reported in monotherapy arm), without any apparent gain in efficacy [60]. These results suggest that adding tremelimumab to PD-L1 blockade does not meaningfully augment responses in an unselected, predominantly pMMR population.

#### 4.3.2. Ipilimumab

Ipilimumab is a monoclonal antibody targeting CTLA-4, established in other solid tumors in combination with nivolumab. In EC, published EC-specific efficacy data are absent from the available literature. In NRG-GY025 Ongoing Trials, previously mentioned in the section addressing nivolumab and featured in Table 4, ipilimumab is being investigated alongside nivolumab in dMMR EC. A phase Ib trial examined novel intraperitoneal delivery of combination ipilimumab and nivolumab (NCT03508570) and demonstrated safety of the regimen, though this cohort was predominantly ovarian cancer, with only 2 out of 23 patients with uterine cancer [61].

### 4.4. Emerging Checkpoint Targets

Beyond the established PD-1, PD-L1, and CTLA-4 axes, several additional co-inhibitory receptors are expressed in the EC TME and represent potential targets for next-generation checkpoint blockade. No agents in this category have yet demonstrated efficacy in EC-specific clinical trials, and the discussion below reflects early-phase or pre-clinical investigation.

Lymphocyte-activation gene 3 (LAG-3) is a co-inhibitory receptor expressed on activated T and NK cells. It suppresses CD4+ T-cell activation primarily through binding to major histocompatibility complex (MHC) class II molecules on antigen-presenting cells. More recently, fibrinogen-like protein 1, secreted at high levels by tumor cells, has been identified as a key LAG-3 ligand capable of directly suppressing CD8+ T-cell activation [26]. Given the high neoantigen burden and T-cell infiltration characteristic of dMMR EC, LAG-3 is an attractive target in this molecular subtype and could theoretically synergize with PD-(L)1 blockade. Existing anti-LAG-3 antibodies include relatlimab, favezelimab, and ieramilimab; the combination of nivolumab and relatlimab is FDA approved for advanced melanoma [62]. The anti-LAG-3 antagonist antibody INCAGN02385 was evaluated in a phase I dose escalation study (NCT03538028) that enrolled 22 patients with advanced solid tumors, none of whom experienced a response and six of whom achieved stable disease. The cohort included two patients with EC, one of whom achieved stable disease [62].

B7-H4 is a co-inhibitory molecule of the B7 family expressed on TAMs and tumor cells that suppresses T-cell proliferation, cytokine secretion, and cell cycle progression. It has been detected in a high proportion of EC tissues, with expression patterns that may correlate with tumor aggressiveness [26]. The anti-B7-H4 antibody FPA150 was investigated in a phase Ia/b study (NCT03514121) in advanced solid tumors including EC, with no endometrial-specific results yet available [26].

TIM-3 (T-cell immunoglobulin and mucin-domain containing-3) is a co-inhibitory receptor expressed on exhausted CD4+ and CD8+ T cells, Tregs, NK cells, and antigen-presenting cells, acting through a mechanism distinct from the PD-1 axis. Focal TIM-3 expression has been demonstrated in all EC cases examined in one series, with substantially stronger expression in dMMR compared with pMMR tumors, suggesting particular relevance in molecularly immunogenic subtypes. No clinical trials specifically targeting TIM-3 in EC have been reported in the literature to date [26].

Other emerging targets referenced in the context of EC include TIGIT (T-cell immunoreceptor with immunoglobulin and ITIM domains) and poliovirus receptor-related immunoglobulin domain-containing protein (PVRIG; an NK cell checkpoint protein), both of which are under evaluation in early-phase combination studies with PD-(L)1 inhibitors [63]. These represent early-stage hypotheses rather than clinically validated strategies in EC.

### 4.5. Immune-Cold Endometrial Cancer

Immune-cold tumors are characterized by sparse T-cell infiltration, low PD-L1 expression, and limited TMB, in contrast to immune-inflamed or “hot” tumors, which are the most responsive to ICIs [25]. Cold tumors encompass both immune-excluded and immune-desert phenotypes, which typically require combination strategies that remodel the TME to permit immune cell entry and activity [22]. In EC, NSMP and p53abn tumors align most closely with this phenotype (Section 2.5) [29]. However, a subset of dMMR tumors also lacks meaningful immune infiltration despite high TMB, indicating that MMR status and immune contexture are not interchangeable [22].

Beyond a low neoantigen burden, tumor-intrinsic oncogenic signaling actively shapes immune exclusion. Wnt/β-catenin pathway activation through CTNNB1 exon 3, APC, or SOX17 alterations is frequent in NSMP tumors. It impairs effector T-cell function and appears to be a major driver of immune exclusion [14,20]. PTEN loss further reduces interferon signaling while increasing Treg density and immunosuppressive cytokine production [22]. Downregulation of MHC class I impairs antigen presentation, and IDO1-mediated kynurenine production suppresses effector T-cell function. Together with the VEGF- and myeloid-driven mechanisms described in Section 3, these alterations sustain a profoundly immune-evasive microenvironment [24,30].

Current therapeutic strategies seek to convert cold tumors into inflamed ones. Anti-angiogenic agents reduce Treg activity, reverse VEGF-mediated immunosuppression, and improve CD8+ T-cell trafficking into the tumor bed [3,20]. This is the most clinically validated approach in pMMR disease, as demonstrated by lenvatinib–pembrolizumab and avelumab–axitinib (Section 4.1.1 and Section 4.2.2). Chemotherapy and radiotherapy induce immunogenic cell death, thereby enhancing tumor antigen presentation and immune infiltration [25]. Chemotherapy–ICI combinations are now established in the first-line setting, though with more modest benefit in pMMR disease. Radiotherapy may additionally elicit systemic abscopal responses, although EC-specific data in combination with ICIs remain immature [24].

ADCs represent a newer partner for checkpoint blockade. Their cytotoxic payloads induce immunogenic cell death and dendritic cell maturation. Payload diffusion to neighboring antigen-negative cells, the so-called bystander effect, is particularly relevant given the heterogeneous antigen expression characteristic of EC [64,65]. In a pre-clinical HER2-positive model with primary resistance to checkpoint blockade, trastuzumab emtansine (T-DM1) rendered tumors curable with combined CTLA-4/PD-1 inhibition [66]. Clinically, mirvetuximab soravtansine plus pembrolizumab has shown activity in pMMR serous EC (Section 4.1.1), and phase III trials are ongoing (Section 5). Finally, dual-checkpoint blockade targets complementary steps of T-cell priming and effector function (Section 3.4). However, the addition of tremelimumab to durvalumab failed to improve responses in a predominantly pMMR population, and next-generation bispecific antibodies co-targeting PD-1 with VEGF or TIGIT are now under investigation (Section 5) [60].

No validated biomarker yet identifies which immune-cold tumors will respond to a given combination, and integrated immune profiling beyond the MMR status will be essential to guide rational patient selection [22].

## 5. Ongoing Trials

The clinical development landscape for ICIs in EC continues to expand rapidly, with trials addressing unresolved questions across disease settings, biomarker-selected populations, and novel combination strategies. Key elements of select ongoing trials examining ICIs in EC are shown in Table 4.

In the first-line dMMR/MSI-H setting, two phase III trials are testing ICI monotherapy as an alternative to chemotherapy. KEYNOTE-C93/GOG-3064/ENGOT-en15 (NCT05173987) is randomizing 280 patients to pembrolizumab monotherapy versus carboplatin–paclitaxel, while DOMENICA/ENGOT-en13 (NCT05201547) is evaluating dostarlimab monotherapy versus chemotherapy in 260 patients, with crossover to dostarlimab permitted at progression. Both trials use PFS as their primary endpoint and are currently active but no longer enrolling; results will determine whether chemotherapy can be omitted entirely in this molecular subgroup.

For first-line pMMR disease, where the benefit of adding ICI to chemotherapy is more modest, several trials are testing maintenance intensification strategies. TroFuse-033/GOG-3119/ENGOT-en29 (NCT06952504) is a phase III trial randomizing 1123 patients after induction carboplatin–paclitaxel–pembrolizumab to either pembrolizumab maintenance alone or sacituzumab tirumotecan (a trophoblast cell-surface antigen 2 (TROP-2)-directed ADC) plus pembrolizumab, with co-primary endpoints of PFS and OS. The phase II PENELOPE trial (NCT06502743) is exploring the addition of nesuparib, a PARP1/2 and tankyrase inhibitor, to pembrolizumab maintenance. Symbiotic-GYN-18 (NCT07578649) will directly compare a bispecific PD-1/VEGF antibody (PF-08634404) plus chemotherapy against pembrolizumab plus chemotherapy in pMMR patients.

Neoadjuvant and fertility-sparing strategies are an emerging frontier. The SATELLITE trial (NCT06278857) is evaluating dostarlimab as organ-sparing treatment in patients with stage I dMMR endometrioid EC who are ineligible for or declining surgery, with a pathologic complete response as the primary endpoint. Several additional single-arm phase II studies are examining pre-operative ICI in dMMR disease, including neoadjuvant pembrolizumab (NCT06180733) and neoadjuvant dostarlimab (GEICO 137-E trial, NCT07013851 and RODEO trial, NCT07115927).

In the recurrent setting, NRG-GY025 (NCT05112601) is a randomized phase II trial comparing nivolumab monotherapy to nivolumab plus ipilimumab in dMMR recurrent EC, addressing whether dual checkpoint blockade offers an incremental benefit. SERPENTINE (NCT06680739) is evaluating durvalumab with or without a single priming dose of tremelimumab across MSI and MSS tumor populations. For pMMR recurrent disease following prior ICI exposure—a clinically challenging scenario with no established standard—a dedicated phase II trial (NCT07594015) is examining the re-deployment of pembrolizumab–lenvatinib after first-line chemotherapy containing immunotherapy. The phase III fruquintinib–sintilimab trial (NCT06584032) is evaluating an oral VEGFR inhibitor combined with an anti-PD-1 antibody versus chemotherapy in 412 pMMR patients in the second-line setting.

Beyond the trials represented in Table 4, a broader portfolio of early-phase studies is exploring novel ICI combination partners in EC, including ADCs like fam-trastuzumab deruxtecan-nxki (Enhertu, NCT06989112) and BL-B01D1, a bispecific ADC against epidermal growth factor receptor (EGFR) and human epidermal growth factor receptor 3 (HER3) (NCT07054567). ICI pairings with bispecific antibodies against mucin 16 (MUC16)/CD28 (NCT04590326) and αvβ8/αvβ1 integrins (NCT06270706) are being examined. Additional emerging ICI therapies against novel targets include agents directed against immunoglobulin superfamily member 8 (IGSF8, NCT05669430), signal-regulatory protein alpha (SIRPα, NCT05856981), leukocyte-associated immunoglobulin-like receptor-1 (LAIR-1, NCT05572684), and TIGIT (NCT05277051), alongside metabolic and DNA damage response combinations pairing ICIs with IDO1 inhibitors (NCT04106414), ataxia telangiectasia and Rad3-related protein serine/threonine kinase (ATR) inhibitors (NCT03682289 and NCT04065269), poly(ADP-ribose) glycohydrolase (PARG) inhibitors (NCT05787587), and Werner helicase inhibition (NCT07262619). An in-depth exploration of the rationale and study design of each trial is beyond the scope of this review. While individually early-stage, these trials collectively reflect a shift toward precision immunotherapy combinations tailored to specific immune evasion mechanisms operative in molecularly defined EC subgroups.

## 6. Conclusions

Immune checkpoint inhibition has fundamentally reshaped the management of advanced and recurrent EC. What was once a disease with limited systemic options beyond platinum-based chemotherapy has become one of the most therapeutically active areas in oncology, with multiple approved ICI-based regimens now spanning first- and second-line settings.

The most robust gains have been achieved in patients with dMMR/MSI-H disease. Single-agent PD-1 blockade in platinum-pretreated patients established the principle of durable, biomarker-selected responses, and subsequent phase III trials have demonstrated that incorporating ICI into first-line chemotherapy, with maintenance ICI thereafter, produces large and clinically meaningful improvements in PFS and OS in this molecular subset. These results have made MMR testing mandatory in advanced EC and elevated ICI-chemotherapy combinations to standard of care. The lenvatinib–pembrolizumab combination has similarly become an established option in the second-line setting regardless of MMR status, extending the ICI benefit to patients with pMMR disease. Collectively, these advances have made ICI therapy a cornerstone of EC treatment across multiple lines.

Nonetheless, important unmet needs remain. The benefit of ICI in pMMR/MSS disease, which constitutes the majority of advanced EC cases, remains modest in magnitude and lacks reliable predictive biomarkers beyond MMR status. The adjuvant setting has proven more challenging, with ICI added to adjuvant chemotherapy failing to improve outcomes in the overall population despite signals of benefit in dMMR subgroups. Optimal treatment sequencing after prior ICI exposure is largely uncharted, with ongoing trials exploring this knowledge gap. For the most aggressive EC subtypes with cold immune microenvironments, the current generation of checkpoint inhibitors provides limited benefit as monotherapy.

The ongoing trial landscape reflects efforts to address these gaps on multiple fronts. Phase III trials are evaluating ICI monotherapy as a potential replacement for chemotherapy in first-line dMMR disease, while intensified maintenance strategies combining ICI with ADCs or PARP inhibitors are under investigation for pMMR patients. Neoadjuvant ICI approaches are emerging as a novel frontier, with potential implications for organ preservation and fertility-sparing treatment. Beyond established PD-1/PD-L1 and CTLA-4 axes, early-phase studies are exploring novel checkpoint targets, bispecific antibodies, and rational ICI combinations with DNA damage response agents and metabolic pathway inhibitors, reflecting a broader shift toward precision immunotherapy matched to specific immune evasion mechanisms.

In summary, immune checkpoint inhibitors have become integral to the treatment of endometrial carcinoma, delivering survival benefits that were not achievable with conventional therapy. The field continues to evolve rapidly, and ongoing research into biomarker refinement, rational combination strategies, and novel therapeutic targets will be essential to extend these benefits to the full breadth of patients with this disease.

## Figures and Tables

**Table 1:** Molecular classification of endometrial carcinoma.

Molecular Subtype	Molecular Definition	Prevalence	Additional Associations	Prognosis
**POLE-Mutated** (*POLEmut*) TCGA: POLE Ultramutated	Pathogenic somatic mutations in the POLE exonuclease domain abolish polymerase proofreading fidelity Ultramutated phenotype (TMB >100 mut/Mb), the highest of all EC subtypes	**~7–10%**	**Histology/TILs:** Predominantly high-grade endometrioid; dense CD8+ TIL infiltration reflects extreme tumor immunogenicity driven by massive neoantigen burden, underpinning the favorable prognosis despite adverse histologic features	**EXCELLENT**
**MMR-Deficient** (*MMRd*) TCGA: MSI Hypermutated	Loss of MMR protein expression (MLH1, MSH2, MSH6, and/or PMS2) by IHC, most commonly via somatic MLH1 promoter hypermethylation Results in dMMR/MSI-H and second-highest TMB among EC subtypes (10–100 mut/Mb)	**~25–28%**	**Lynch Syndrome:** ~15–17% of dMMR ECs harbor germline MMR mutations; universal MMR/MSI screening is recommended for all newly diagnosed ECs to identify Lynch syndrome probands and direct cascade genetic testing	**INTERMEDIATE**
**No Specific Molecular Profile** (*NSMP*) TCGA: Copy Number Low	Diagnosis of exclusion: absence of *POLE* mut, intact MMR expression, and wild-type p53 IHC Enriched for PTEN, PIK3CA, CTNNB1, and KRAS mutations with low TMB, low SCNAs, and predominantly ER-positive, MSS profile	**~40–50%**	**Histology:** Most heterogeneous subtype; predominantly low-grade endometrioid, but also encompasses clear-cell and dedifferentiated carcinomas; CTNNB1 exon 3 mutations identify a subset with paradoxically elevated recurrence risk despite low-grade histology	**INTERMEDIATE**
**p53-Abnormal** (*p53abn*) TCGA: Copy Number High	*TP53* mutations in >90% of cases detected by IHC as aberrant p53 expression (overexpression, null, or cytoplasmic pattern) Associated with extensive SCNAs and low TMB	**~12–20%**	**Histology:** Encompasses most serous carcinomas and carcinosarcomas, and a subset of high-grade endometrioid **Mortality:** Accounts for >50% of EC-related mortality despite representing 12–20% of cases **HER2:** ~20–25% overexpress HER2, representing an additional treatment target	**POOR**

**Abbreviations:** EC, endometrial carcinoma; ER, estrogen receptor; IHC, immunohistochemistry; Mb, megabase; MMRd, mismatch repair-deficient; MSI, microsatellite instability; MSI-H, microsatellite instability-high; MSS, microsatellite-stable; mut, mutations; NSMP, no specific molecular profile; SCNA, somatic copy number alteration; TCGA, The Cancer Genome Atlas; TIL, tumor-infiltrating lymphocyte; TMB, tumor mutational burden. **Note:** Molecular subtype assignment follows a hierarchical algorithm: POLE sequencing first, followed by MMR IHC, then p53 IHC, with NSMP assigned when all three are negative or normal. Approximately 3–6% of cases harbor more than one molecular alteration (“multiple classifiers”); POLE mutation confers the dominant prognostic effect when co-occurring with p53abn or MMRd.

**Table 2:** FDA-approved immune checkpoint inhibitor indications in endometrial cancer.

ICI (Brand Name)	Regimen	Approved Setting	Biomarker	Trial Name	NCT No.	Approval Month and Year
**Pembrolizumab** (Keytruda)	Pembrolizumab monotherapy	Advanced/recurrent/metastatic	dMMR/MSI-H	KEYNOTE-158	NCT02628067	Tumor agnostic approval May 2017; EC specific approval March 2022
	Pembrolizumab + lenvatinib	Advanced/recurrent after front-line chemotherapy	pMMR	KEYNOTE-775/Study 309	NCT03517449	Accelerated approval September 2019 based on KEYNOTE-146 Full approval July 2021
	Pembrolizumab + carboplatin–paclitaxel → pembrolizumab maintenance	Front-line advanced (stage III/IV) or recurrent	All-comers	KEYNOTE-868/NRG-GY018	NCT03914612	June 2024
**Dostarlimab-gxly** (Jemperli)	Dostarlimab monotherapy	Advanced/recurrent after front-line chemotherapy	dMMR/MSI-H	GARNET	NCT02715284	Accelerated approval April 2021
	Dostarlimab + carboplatin–paclitaxel → dostarlimab maintenance	Front-line advanced (stage III/IV) or recurrent	All-comers	RUBY/ENGOT-en6	NCT03981796	Initial approval dMMR only July 2023; expanded to all patients August 2024
**Durvalumab** (Imfinzi)	Durvalumab + carboplatin–paclitaxel → durvalumab maintenance	Front-line advanced (stage III/IV) or recurrent EC	dMMR	DUO-E/GOG-3041	NCT04269200	June 2024

**Abbreviations:** dMMR, mismatch repair-deficient; MSI-H, microsatellite instability-high; pMMR, mismatch repair-proficient.

**Table 3:** Summary of concluded clinical trials of immune checkpoint inhibitors in endometrial cancer.

Trial Name (NCT No.)	Phase	Trial Arms	Treatment Setting (Stage/Prior Lines)	Biomarker Eligibility	Primary Endpoint(s) and Key Efficacy Results	FDA Approval Status
**PD-1 Inhibitors: Pembrolizumab, Nivolumab, Dostarlimab**						
KEYNOTE-158 (NCT02628067)	II	Pembrolizumab monotherapy (single-arm)	Advanced/recurrent EC (unresectable/metastatic) Failure or intolerance to standard therapies	MSI-H/dMMR (required)	**Primary:** ORR **ORR:** 48% (95% CI 37–60) **CR:** 14% **Median PFS:** 13.1 mo **Median OS:** NR **3-yr OS:** ~60%	**Approved** dMMR/MSI-H advanced EC after prior therapy
KEYNOTE-868/ NRG-GY018 (NCT03914612)	III	Pembrolizumab + CP → pembrolizumab maintenance vs. Placebo + CP → placebo	1L advanced (stage III/IV) or recurrent EC except carcinosarcoma No prior systemic chemo for recurrence	All-comers (stratified by MMR status)	**Primary:** PFS (per cohort) **dMMR—12-mo PFS:** 74% vs. 38% (HR 0.30; *p* < 0.001) **pMMR—Median PFS:** 13.1 vs. 8.7 mo (HR 0.54; *p* < 0.001)	**Approved** All-comer advanced or recurrent EC in combination with CP
KEYNOTE-B21/ ENGOT-en11 (NCT04634877)	III	Pembrolizumab + CP ± RT → pembrolizumab maintenance vs. placebo + CP ± RT → placebo	Adjuvant; high-risk EC defined as early stage + non-endometrioid or p53 mutant, or stage III/IVA No measurable residual disease	All-comers (stratified by MMR status)	**Primary:** DFS and OS **ITT—DFS:** HR 1.02 (95% CI 0.79–1.32; *p* = 0.570)—not met **dMMR subgroup:** HR 0.31 (95% CI 0.14–0.69) **pMMR subgroup:** HR 1.20 (95% CI 0.91–1.57) **OS:** Awaiting maturation	**Not approved** in all-comer adjuvant treatment
KEYNOTE-775/ Study 309 (NCT03517449)	III	Lenvatinib + pembrolizumab vs. physician’s choice chemo (doxorubicin or paclitaxel)	Advanced/recurrent EC 1 prior platinum-based regimen No prior anti-PD-(L)1	All-comers (stratified by MMR status)	**Primary:** PFS and OS **pMMR—Median OS:** 18.0 vs. 12.2 mo (HR 0.70; 95% CI 0.60–0.83) **dMMR—Median OS:** 31.9 vs. 8.6 mo (HR 0.44; 95% CI 0.29–0.67) **5-yr OS (all-comers):** 19.9% vs. 7.7%	**Approved** for pMMR/MSS advanced EC after prior platinum
LEAP-001/ ENGOT-en9 (NCT03884101)	III	Lenvatinib + pembrolizumab vs. CP	1L stage III–IV or recurrent EC Prior platinum permitted if neo/adjuvant (PFI ≥ 6 months)	All-comers (stratified by MMR status)	**Primary:** PFS and OS **pMMR—Median PFS:** 9.6 vs. 10.2 mo (HR 0.99)—not met **pMMR—Median OS:** 30.9 vs. 29.4 mo (HR 1.02; non-inferiority *p* = 0.246)—not met	**Not approved** in 1L setting
NCI-MATCH Arm Z1D (NCT02465060)	II	Nivolumab monotherapy (single-arm sub-study)	Recurrent advanced solid tumors, including EC No limit on prior therapies (median 3 lines)	dMMR (loss of MLH1 or MSH2 IHC)	**Primary:** ORR Activity confirmed in dMMR non-CRC cancers including EC Overall ORR 36%—**endometrial-specific ORR not separately published**	**Not approved**
Lheureux et al. Phase II (NCT03367741)	II	Arm A: Cabozantinib + nivolumab Arm B: Nivolumab monotherapy Arm C: Cabozantinib + nivolumab (post-ICI or carcinosarcoma)	Advanced/recurrent EC No limit on prior therapy; at least one prior line Prior ICI permitted only in Arm C	All-comers (stratified by MMR status)	**Primary:** PFS **Median PFS (A vs. B):** 5.3 vs. 1.9 mo (HR 0.59; *p* = 0.09) **ORR (A vs. B):** 25% vs. 11% **Arm C ORR:** 25% (10% for carcinosarcoma subgroup)	**Not approved**
GARNET (NCT02715284)	I	Dostarlimab monotherapy (single-arm)	Advanced/recurrent EC after platinum-based doublet chemo ≤2 prior lines permitted	Biomarker-selected (dMMR and pMMR cohorts)	**Primary:** ORR, DOR **dMMR ORR:** 45.5% (95% CI 37.1–54.0) **dMMR CR:** 16.1% **Median dMMR DOR:** NR (83.7% maintained response at 24 mo) **pMMR ORR:** 15.4%	**Approved** for dMMR recurrent/advanced EC after platinum
RUBY/ ENGOT-en6 (NCT03981796)	III	Dostarlimab + CP → dostarlimab maintenance vs. placebo + CP → placebo	1L primary advanced (stage III/IV) or first-recurrent EC Included carcinosarcoma	All-comers (stratified by MMR status)	**Primary:** PFS and OS **dMMR—24-mo PFS:** 61.4% vs. 15.7% (HR 0.28; 95% CI 0.16–0.50) **dMMR—24-mo OS:** 83.3% vs. 58.7% (HR 0.30; 95% CI, 0.13–0.70) **Overall—24-mo PFS:** 36.1% vs. 18.1% (HR 0.64; 95% CI, 0.51–0.80) **Overall—24-mo OS:** 71.3% vs. 56.0% (HR 0.64; 95% CI 0.46–0.87)	**Approved** for primary advanced or recurrent EC in combination with CP, regardless of MMR status
**PD-L1 Inhibitors: Atezolizumab, Avelumab, Durvalumab**						
AtTEnd (NCT03603184)	III	Atezolizumab + CP → atezolizumab maintenance vs. placebo + CP → placebo	1L advanced or recurrent EC/carcinosarcoma Prior platinum permitted if neo/adjuvant (PFI ≥ 6 months)	All-comers (stratified by MMR status)	**Primary:** PFS and OS **dMMR—Median PFS:** NR vs. 6.9 mo (HR 0.36; 95% CI 0.23–0.57) **Overall—Median PFS:** 10.1 vs. 8.9 mo (HR 0.74; 95% CI 0.61–0.91) **Interim median OS (overall):** 38.7 vs. 30.2 mo (HR 0.82; OS boundary not crossed)	**Not approved**
Konstantinopoulos et al.; Lee et al. Phase II (3 cohorts) (NCT02912572)	II	Cohort 1: Avelumab monotherapy (dMMR and pMMR)	Recurrent/persistent EC (any histology) One or more prior chemo lines (no upper limit), no prior ICI	dMMR cohort (primary) pMMR cohort (closed early—futility)	**Primary:** ORR and PFS6 **dMMR ORR:** 26.7% (95% CI 7.8–55.1) **dMMR PFS6:** 40.0% (95% CI 16.3–66.7) **pMMR:** Closed after stage 1; ORR 6.25%	**Not approved** (Phase II only)
		Cohort 2: Avelumab + talazoparib	Recurrent/persistent EC (any histology) One or more prior chemo lines (no upper limit), no prior ICI	pMMR required	**Primary:** ORR and PFS6 **ORR:** 11.4% (95% CI 3.2–26.7) **PFS6:** 22.9%; median PFS 3.6 mo **Subset with HRD and/or PFI ≥ 6 mo:** ORR 25%, PFS6 58.3%, median PFS 8 mo	**Not approved** (Phase II only)
		Cohort 3: Avelumab + axitinib	Recurrent/persistent EC (any histology) One or more prior chemo lines (no upper limit), no prior ICI	pMMR required	**Primary:** ORR and PFS6 **Confirmed ORR:** 25.7% (95% CI 12.5–43.3) **Median DOR:** 13.8 mo; **PFS6:** 55.8%; median PFS 7 mo **TP53mut/NSMP subsets:** ORR 48% vs. 28.6%	**Not approved** (Phase II only)
PHAEDRA (ANZGOG1601; NCT03015129)	II	Durvalumab monotherapy (single arm)	Advanced/recurrent EC, except carcinosarcoma 1–3 prior lines if pMMR, 0–3 prior lines if dMMR, no prior ICI	Biomarker-selected (dMMR and pMMR cohorts)	**Primary:** ORR **dMMR ORR:** 47% (95% CI 32–63); 6 CR **dMMR Median PFS:** 8.3 mo **dMMR 12-mo OS**: 71% **pMMR ORR:** 3% (95% CI 1–15) **pMMR median PFS:** 1.8 mo	**Not approved**
DUO-E (GOG-3041; NCT04269200)	III	Arm 1 (control): Placebo + CP → placebo maintenance Arm 2: Durvalumab + CP → durvalumab maintenance Arm 3: Durvalumab + CP → durvalumab + olaparib maintenance	1L newly diagnosed advanced or recurrent EC except sarcomas Prior chemo permitted if adjuvant (disease-free ≥ 12 months)	All-comers (stratified by MMR status)	**Primary:** PFS (Arm 2 vs. 1; Arm 3 vs. 1) **Arm 2 vs. control—PFS HR:** 0.71 (95% CI 0.57–0.89) **Arm 3 vs. control—PFS HR:** 0.55 (95% CI 0.43–0.69) **Interim OS:** Directionally supportive; neither comparison met significance **Exploratory Arm 3 vs. Arm 2—PFS HR:** 0.78 (95% CI 0.61–0.99), benefit driven by pMMR cohort	**Approved** in combination with CP for dMMR primary advanced/recurrent EC **Not approved** as combination maintenance therapy with olaparib
**CTLA-4 Inhibitors: Tremelimumab**						
Rubinstein et al. Phase II (NCT03015129)	II	Arm 1: Durvalumab monotherapy Arm 2: Durvalumab + tremelimumab	Persistent or recurrent EC, including carcinosarcoma After 1–4 prior lines	All-comers (stratified by MMR status)	**Primary:** ORR and PFS at 24 wks **Arm 1 ORR:** 10.8% **Arm 2 ORR:** 5.3% **Median PFS (both arms):** ~7–8 weeks Primary endpoint not met in either arm	**Not approved**
***Additional Combinations***						
Fuh et al. Phase II (NCT03526432)	II	Atezolizumab + bevacizumab (single-arm)	Recurrent EC Prior platinum-based chemo required, no prior ICI	All-comers	**Primary:** ORR **ORR:** 33.9% (95% CI 21.8–47.8) **Median DOR:** 16 mo **Median PFS:** 8.8 mo (95% CI 5.5–12.0) **Median OS:** 41.55 mo (95% CI 27.0–57.5) ⚠ Meeting abstract only (ASCO 2026 [38]); data preliminary	**Not approved** (Phase II; data preliminary)
Porter et al. Phase II (NCT03835819)	II	Mirvetuximab soravtansine + pembrolizumab (single-arm)	Recurrent/persistent serous EC 1–4 prior lines of therapy, prior ICI allowed	pMMR/MSS and FOLR1 ≥2+ staining in ≥50% cells (required)	**Primary:** ORR and PFS6 **Confirmed ORR:** 28% (95% CI 10–53) **PFS6:** 24.4% (95% CI 7.7–46.1) **Median DOR:** 8.17 mo Closed early, met prespecified feasibility endpoint of ≥4 ORR (18/35 planned)	**Not approved** (Phase II, data preliminary)

Abbreviations: CP; carboplatin–paclitaxel; dMMR, mismatch repair-deficient; DFS, disease-free survival; DOR, duration of response; EC, endometrial cancer/carcinoma; FOLR1, folate receptor alpha; HR, hazard ratio; ICI, immune checkpoint inhibitor; ITT, intention-to-treat; 1L, first-line; mo, months; MMR, mismatch repair; NR, not reached; ORR, objective response rate; OS, overall survival; PFI, platinum-free interval; PFS, progression-free survival; pMMR, mismatch repair-proficient; RT, radiotherapy; wks, weeks. ⚠ Fuh et al. data are from a meeting abstract (ASCO 2026 [38]) and should be considered preliminary pending full peer-reviewed publication.

**Table 4:** Ongoing clinical trials of immune checkpoint inhibitors in endometrial cancer.

Trial Name (NCT No.)	Phase	Trial Arms	Treatment Setting (Stage/Prior Lines)	Target Enrolment	Biomarker Selection	Primary Endpoint(s)	Recruitment Status	Est. Completion Date
**PD-1 Inhibitors: Pembrolizumab, Nivolumab, Dostarlimab**								
**KEYNOTE-C93/GOG-3064/ENGOT-en15** NCT05173987	III	**Arm A:** Pembrolizumab (up to 18 cycles) **Arm B:** Carboplatin + paclitaxel × 6 cycles	1L advanced (stage III/IV) or recurrent (including carcinosarcoma) No prior systemic therapy permitted, except for adjuvant or NACT	280	dMMR/MSI-H	**Primary:** PFS, OS	Active, not recruiting	May 2027
**DOMENICA/GINECO-EN105b/ENGOT-en13** NCT05201547	III	**Arm A:** Dostarlimab **Arm B:** Carboplatin + paclitaxel X 6 cycles (crossover to dostarlimab at progression permitted)	1L advanced or recurrent, any histology No prior systemic therapy permitted, except for adjuvant or NACT	260	dMMR/MSI-H	**Primary:** PFS **Key secondary:** OS	Active, not recruiting	April 2026
**Symbiotic-GYN-18** NCT07578649	III	**Arm A:** PF-08634404 (bispecific PD-1/VEGF antibody) + carboplatin–paclitaxel → PF-08634404 maintenance **Arm B:** Pembrolizumab + carboplatin–paclitaxel → pembrolizumab maintenance	1L advanced or recurrent pMMR EC No prior systemic therapy permitted, except for adjuvant or NACT	600	pMMR	**Primary:** PFS	Not yet recruiting	June 2029
**TroFuse-033/GOG-3119/ENGOT-en29 (MK-2870-033)** NCT06952504	III	**Induction (all arms):** Carboplatin + paclitaxel + pembrolizumab × 6 cycles **Maintenance Arms:** Sacituzumab tirumotecan (MK-2870; TROP-2-directed ADC) + pembrolizumab vs. MK-2870 alone vs. pembrolizumab alone	1L advanced or recurrent pMMR EC No prior systemic therapy permitted, except for adjuvant or NACT	1123	pMMR	**Primary:** PFS, OS	Recruiting	May 2032
**NRG-GY020** NCT04214067	III	**Arm A:** Pembrolizumab + adjuvant radiotherapy (vaginal brachytherapy ± EBRT) **Arm B:** Adjuvant radiotherapy alone	Adjuvant; newly diagnosed stage I–II high-intermediate risk endometrioid EC No measurable residual disease post-surgery	164	dMMR, endometrioid histology	**Primary:** 3-year recurrence-free survival	Active, not recruiting	December 2025
**PENELOPE** NCT06502743	II	**Induction (all):** Carboplatin–paclitaxel + pembrolizumab × 6 cycles **Maintenance Arm A:** Pembrolizumab + nesuparib (PARP1/2 and tankyrase 1/2 inhibitor) **Maintenance Arm B:** Pembrolizumab monotherapy	1L advanced or recurrent pMMR EC Prior adjuvant treatment, HT, and RT allowed	92	pMMR	**Primary:** Part A: DLT rate (feasibility); Part B: PFS	Recruiting	December 2027
**Pembrolizumab + Lenvatinib in Post-ICI pMMR Recurrent EC** NCT07594015	II	**Single-arm:** Pembrolizumab + lenvatinib	Recurrent pMMR EC ≥1 prior platinum-based line + ICI required	46	pMMR	**Primary:** Clinical benefit rate	Recruiting	June 2031
**Pembrolizumab + Lenvatinib in Advanced Uterine Carcinosarcoma (MSK)** NCT05147558	II	**Single-arm:** Pembrolizumab + lenvatinib	Advanced or recurrent uterine or ovarian carcinosarcoma 1–3 prior lines, ICI-naïve	40	Unselected (MMR not required)	**Primary:** ORR, PFS	Active, not recruiting	December 2026
**Pembrolizumab + Lenvatinib + Paclitaxel in Recurrent EC/Ovarian Cancer** NCT04781088	II	**Single-arm:** Pembrolizumab + lenvatinib + weekly paclitaxel	Recurrent EC (and platinum-resistant ovarian/fallopian tube/peritoneal carcinoma) 1–3 prior lines, prior ICI allowed	38	Unselected (MMR not required)	**Primary:** ORR	Active, not recruiting	July 2025
**AdHER2DC Vaccine + Pembrolizumab + Lenvatinib + N-803** NCT06253494	I/II	**Phase I:** Dose escalation—AdHER2DC vaccine (HER2-targeting dendritic cell vaccine) + N-803 (IL-15 superagonist) + pembrolizumab + lenvatinib **Phase II:** Expansion at recommended ph 2 dose	Advanced or metastatic EC ≥1 prior platinum-based line	60	HER2 IHC 1+, 2+, or 3+	**Primary:** Phase I: DLTs/RP2D; Phase II: PFS at 6 months	Recruiting	December 2026
**Neoadjuvant Pembrolizumab in dMMR EC (PAM-II)** NCT06180733	II	**Single-arm:** Pembrolizumab × 9 cycles prior to planned hysterectomy	Newly diagnosed resectable dMMR EC, grade 3 or clear cell Neoadjuvant; treatment-naïve Patients eligible for hysterectomy	12	dMMR	**Primary:** Major pathological response rate at hysterectomy (~6 months)	Active, not recruiting	December 2027
**Preoperative Dostarlimab and Novel Therapies in EC (RODEO)** NCT07115927	II	**Single-arm:** Dostarlimab (pre-operative) × 2 cycles followed by surgery	Newly diagnosed operable stage II–IV EC except carcinosarcoma Neoadjuvant; treatment-naïve	40	Unselected (MMR status stratified; all-comer)	**Primary:** Pathological response rate at time of surgery	Not yet recruiting	April 2030
**Neoadjuvant Dostarlimab in dMMR/MSI-H Stage II–III EC (NADIA)** NCT07013851	II	**Single-arm:** Dostarlimab (pre-operative) × 4 cycles followed by surgery and dostarlimab-containing adjuvant therapy	Newly diagnosed stage II–III EC Neoadjuvant; treatment-naïve	25	dMMR/MSI-H, endometrioid histology	**Primary:** Clinical complete response rate	Not yet recruiting	March 2029
**Feasibility, Safety, Erfficacy Dostarlimab Early-Stage Deficient EC (SATELLITE)** NCT06278857	II	**Single-arm:** Dostarlimab × 7 cycles (organ-sparing, non-surgical treatment)	Newly diagnosed stage I dMMR endometrioid EC Patients seeking fertility preservation or unfit/declining surgery	10	dMMR, endometrioid histology	**Primary:** Pathological complete response rate at week 27	Recruiting	June 2026
**NRG-GY025** NCT05112601	II	**Arm A:** Nivolumab monotherapy **Arm B:** Nivolumab + ipilimumab (dual checkpoint blockade) (Safety lead-in precedes randomization)	Recurrent dMMR EC 1–2 prior lines; measurable or non-measurable disease Prior ICI exposure permitted	81	dMMR/MSI-H	**Primary:** PFS (safety lead-in: DLT rate in first 3 cycles)	Recruiting	July 2032
**Fruquintinib + Sintilimab vs. Chemotherapy in Advanced EC** NCT06584032	III	**Arm A:** Fruquintinib (oral VEGFR 1/2/3 inhibitor) + sintilimab (anti-PD-1) **Arm B:** Physician’s choice of chemotherapy (paclitaxel or doxorubicin)	Recurrent EC after failing 1L therapy Prior 1L platinum-based chemo required; not candidates for curative surgery or RT	412	pMMR (required); unselected by PD-L1	**Primary:** PFS, OS	Recruiting	January 2029
**PD-L1 Inhibitors: Atezolizumab, Avelumab, Durvalumab**								
**Avelumab + M1774 in ARID1A-mutated EC** NCT06518564	II	**Single-arm:** Avelumab + M1774 (ATR inhibitor)	Recurrent EC ≥1 prior platinum-based line; prior ICI required	25	ARID1A mutation; MMR not specified	**Primary:** PFS at 6 months, ORR	Recruiting	August 2027
**DOVE (APGOT-OV7/ENGOT-ov80)** NCT06023862	II	**Arm A:** Dostarlimab monotherapy **Arm B:** Dostarlimab + bevacizumab **Arm C:** Non-platinum chemotherapy (liposomal doxorubicin, doxorubicin, gemcitabine, or paclitaxel)	Recurrent gynecological clear-cell carcinoma (ovarian, endometrial, cervical, vaginal, or vulvar) 1–5 lines of prior therapy	198	Clear-cell histology (required); MMR not specified	**Primary:** PFS	Recruiting	August 2027
**Cabozantinib + Dostarlimab in Recurrent Gynecologic Carcinosarcoma** NCT05559879	I/II	**Single-arm:** Cabozantinib + dostarlimab	Recurrent uterine or ovarian carcinosarcoma ≥1 prior platinum-based line	37	Unselected (MMR not required)	**Primary:** PFS at 6 months	Recruiting	April 2027
**ONC201 + Atezolizumab in Obesity-Driven EC** NCT05542407	I	**Non-randomized parallel cohorts:** **Cohort A (obesity-associated EC):** ONC201 (DRD2/3 antagonist and ClpP activator) + atezolizumab **Cohort B (non-obesity-associated EC):** ONC201 + atezolizumab	Advanced or recurrent EC (all epithelial histologies included) At least one prior line of chemotherapy for metastatic disease, or progression within 12 months of adjuvant therapy	58	Unselected (MMR not required)	**Primary:** Recommended phase 2 dose based on DLTs	Recruiting	February 2028
**NRG-GY012** NCT03660826	II	**Arm A:** Durvalumab + olaparib **Arm B:** Cediranib (VEGFR inhibitor) + durvalumab **Arm C:** Olaparib + capivasertib (AKT inhibitor) **Arm D:** Cediranib alone (control) (Earlier phase also tested olaparib alone vs. cediranib alone vs. olaparib + cediranib)	Recurrent, persistent, or metastatic EC (excluding clear-cell and carcinosarcoma) 1–2 prior lines; one prior line of ICI-containing therapy permitted (excluding durvalumab)	288	Unselected (exploratory HRD analyses planned)	**Primary:** PFS	Active, not recruiting	December 2024
**CTLA-4 Inhibitors: Tremelimumab, Ipilimumab**								
**SERPENTINE (Vall d’Hebron Institute of Oncology)** NCT06680739	II	**Arm A:** Durvalumab monotherapy **Arm B:** Tremelimumab single dose 300 mg + durvalumab (MSI EC/CRC) **Arm C:** Tremelimumab single dose 300 mg + durvalumab (MSS EC/CRC)	Recurrent/refractory EC (and CRC) No limits on prior lines of therapy in EC cohort	60	Unselected, but MSI/MMR status must be known for stratification	**Primary:** ORR	Recruiting	July 2032
**Additional Combinations**								
**ATAPEMBRO** NCT04014530	I/II	**Sequential dose escalation then expansion:** **Phase I:** Ataluren (nonsense read-through agent) + pembrolizumab (dMMR and pMMR CRC) **Phase II:** Ataluren + pembrolizumab (dMMR CRC and EC) **Phase II:** Ataluren + pembrolizumab (pMMR CRC)	Metastatic dMMR EC in Phase II; pMMR and dMMR CRC ≥1 prior platinum-based line	47	dMMR required in EC cohort	**Primary:** Phase I: maximum tolerated dose/incidence of treatment-emergent adverse events; Phase II: ORR	Recruiting	June 2023 ⚠
**ROCSAN (ARCAGY/GINECO)** NCT03651206	II/III	**Arm A:** Niraparib monotherapy **Arm B:** Niraparib + dostarlimab **Arm C:** Chemotherapy (physician’s choice)	Recurrent ovarian or endometrial carcinosarcoma ≥1 prior platinum-based line	138	Unselected (carcinosarcoma histology required)	**Primary:** Response rate at 4 months; OS at 1 year after last patient enrollment	Recruiting	June 2027

Abbreviations: ADC, antibody–drug conjugate; ATR, ataxia telangiectasia and Rad3-related protein kinase; ClpP, caseinolytic mitochondrial matrix peptidase proteolytic subunit; CRC, colorectal cancer; DLT, dose-limiting toxicity; dMMR, mismatch repair-deficient; DRD, dopamine receptor; EBRT, external beam radiotherapy; EC, endometrial cancer/carcinoma; HER2, human epidermal growth factor receptor 2; HRD, homologous recombination repair-deficient; HT, hormonal therapy, ICI, immune checkpoint inhibitor; IHC, immunohistochemistry; MMR, mismatch repair; MSI-H, microsatellite instability-high; MSS, microsatellite-stable; NACT, neoadjuvant chemotherapy; ORR, objective response rate; OS, overall survival; PARP, poly(ADP-ribose) polymerase; PD-L(1), programmed death (ligand) 1; PFS, progression-free survival; pMMR, mismatch repair-proficient; RT, radiotherapy; TROP-2, trophoblast cell-surface antigen 2; VEGF, vascular endothelial growth factor; VEGFR, vascular endothelial growth factor receptor; 1L, first-line; 2L, second-line. ⚠ ATAPEMBRO (NCT04014530): Primary completion date listed in clinicaltrials.gov as June 2023. Current recruitment status (listed as ‘Recruiting’ in registry at time of Clinicaltrials.gov query).

## Data Availability

No new data was created during the writing of this narrative review.
